# The Medical College of Ohio

**Published:** 1834

**Authors:** 


### II.—ORIGINAL.

---

### The Western Journal.

---

E Sylvis Nuncius.

---

*Cincinnati, April* 1, 1834.

---

### I. The Medical College of Ohio.

The present number completes the 7th volume of our Journal, and the present month terminates the 7th year since it began. Throughout the whole of this *climacter*, the condition and prospects of the *Medical College of Ohio*, established, as one of its late circulars informs us, in Cincinnati, have been a subject of professional conversation, controversy, and publication. In all that considerable period, moreover, the name of the Editor of this Journal has, in various ways, been associated with the complaints against and from the faculty and trustees of the institution; but his readers will do him the justice to acknowledge, that, with the exception of a few *documents* and mere *notices*, inserted in some of the numbers of the present volume, he has not introduced the subject into its pages. On the contrary, he has generally given publicity to the number of the different classes, the catalogue of graduates, and other items, indicating the progress of the institution.

This forbearance, for which, perhaps, no sufficient reason could at any time be given, has kept the readers of the Journal in ignorance of the policy and progress of the school; when one object of medical journals should be to disseminate correct information concerning medical institutions. The Editor is determined, hereafter, to advise the distant reader, from time to time, of the character and advantages of our Medical College; and to gratify their curiosity as to recent transactions, he proposes to devote a portion of the present number, to the publication and examination of several documents.

As far back as the year 1827, the Third District Medical Society sent a circular to the other Societies of the State, expressing the opinion, that the professorships in the College were not adequately filled. In 1828, the Medical Convention, consisting of delegates from the District Societies, passed a resolution, desiring the General Assembly to do something for the improvement of the School. In the year 1832, the Third District again acted on the subject, and a majority of all its members, by a unanimous vote, a second time directed the attention of the other Districts to the matter, and likewise sent a memorial to the General Assembly. In the following January, 1833, the State Medical Society held its second session, in Columbus, and adopting the resolutions of the Third District, united with them in an application to the Legislature, for a revision and enlargement of the board of Trustees. It was on these joint petitions, that a resolution was passed, authorizing the Governor to appoint a Board of five Commissioners. As already announced in a former number of the Journal, they met at the College edifice, in the month of April last, and made an inquiry.

On the manner of this inquiry two remarks may be made. First. They did not appoint an attorney, or advocate, on the part of the State, nor would they allow the officers of the Society to employ one; while the corporation, embracing seven lawyers, were ably represented by two of them. Secondly. They would not allow testimony to be given to show the general character and standing of the School, compared with other institutions, (clearly the most important part of the inquiry) and in this they conformed to the expressed wishes of the Trustees. Mean while, the law incorporating the District Society, was repealed, dissolving the bodies which had presented the subject to the Legislature, and leaving, of course, no prosecutor. Dr. Martin, however, the President of the Third District Society, and some of its members, who had been a standing Committee on that subject, resolved to step forward, and when the Commissioners were prepared for business, he laid before them the following:

### Charges and Specifications.

*Charges and Specifications preferred against the Trustees of the Medical College of Ohio, at Cincinnati; and the Faculty, or several of them, of the said College, by the Committee of the Third Disirict Medical Society of the State of Ohio.*

### Charge First.

That for several years past the general policy of the Trustees of the Medical College of Ohio, in the city of Cincinnati, has been marked by partiality and prejudice, and indicates an indifference to the reputation and welfare of said College, and to the advance of medical science therein.

*Specifications.*

1st. That in the year 1822, when the said Trustees were appointed, and assumed the charge of the said Medical College, they filled up the vacant Professorships with unknown and incompetent men, to the manifest injury of the character of the institution; and in defiance of public benefit and public opinion, upheld them in office until forced to abandon them by the appearance of a rival institution, in 1831.

2d. That whereas, pupils should have been attracted to the said College by the abilities of its teachers, and the advantages of its circumstances and location alone, the said Trustees, for a period of six or seven years, held the price of tuition lower than in any similar school in the United States.

3d. That in consequence of this undercharging by the said Trustees, the character of the School was degraded abroad, insomuch that the Medical Faculty of Transylvania University, in Kentucky, refused to recognise a course of lectures in the said Medical College of Ohio, as equivalent to one in their own institution; whereby the state of Ohio was injured, and her means of advancing the medical science impaired.

4th. That in the summer of 1831, when the weakness of the said Medical College of Ohio, had invited the establishment of a rival School in the city of Cincinnati, under the auspices of the Miami University, and not until then, the said Trustees displaced three of their professors, created an eighth professorship, to wit, that of Clinical Medicine, and adopted five of the Professors from the said rival school. That Dr. Pierson, a brother-in-law of a member of the Board of Trustees, was retained, and was transferred from the chair of Materia Medica to that of the Institutes of Medicine: that he declined accepting the said chair unless the said Trustees would allow him one year for preparation: that this unjustifiable favor was granted to him by the said Trustees, although he was a citizen of the state of New York, and possessing no peculiar claims upon the said Trustees. That the professorship of Clinical Medicine could not, in the opinion of the class, and of a majority of the Professors, be sustained as a separate chair; but that the chair of the Institutes might have been properly associated with it. That the then professor of Clinical Medicine, who lectured extemporaneously upon the Institutes while the said Dr. Pierson was preparing himself in New York, would willingly have taken the chair formed out of these two, and have continued in the School, but that they adhered to Dr. Pierson, and the former was forced to resign.

5th. That as soon as this was brought about, the said Trustees unwisely abolished both these chairs, and then, in order to bring Dr. Pierson back to the chair of Materia Medica, it was proposed to expel Dr. Henry from the chair of Obstetricks, and give that chair to Dr. Eberle, who refused to accept it: that the said Trustees then adopted a different plan, to wit, they dismissed Dr. Henry from the chair of Obstetrics, transferred Dr. Moorhead to the same chair, from that of Theory and Practice, thus providing a place for Dr. Eberle, whereupon Dr. Pierson was restored to the chair of Materia Medica.

6th. That the person in whose favor all these violent movements were made, was then, and had been from the time of his first appointment, in 1825, a citizen of the state of New York, whence no pupils come to the said Medical College of Ohio; that he was unknown to the physicians of the West, and possessed no peculiar claims upon public attention as a lecturer.

7th. That the gentleman who was compelled to resign, by this adherence of the said Trustees to the interests of Dr. Pierson, had been 43 years in the West; had lectured with the highest approbation to nearly a thousand pupils in the Transylvania University; was on good terms with the Faculy of said Institution, and his talents and services held in great estimation by them; was extensively and advantageously known over the valley of the Mississippi, and indeed throughout the United States; was at this time recommended by the graduates of the Medical College of Ohio for the chair of the Theory and Practice of Medicine, and was the founder of said College, and of the Hospital and Asylum connected with it.

8th. That the gentleman who was improperly expelled, for the purpose of making the aforesaid transfers, that Dr. Pierson who had been eight months preparing himself for the chair of the Institutes, might be brought back to the chair of Materia Medica, was a native of the West; had resided in Kentucky and in Missouri, and was known and esteemed for his talents and general respectability in those states, and also in Illinois and Indiana; that he was at the time of his dismission a permanent citizen of Cincinnati; that at the said time, he had, in the said College, lectured to the decided satisfaction of the class, a majority of whom petitioned against his removal; that he had been one of the professors of the Miami School, which by compromise was united to said College, and that in expelling him without any cause assigned the said Trustees acted unwisely, and unjustly, and violated the faith of said College: That this conduct gave offence to the pupils, especially those from Kentucky, his native state, and created enemies to the College, not only in that state, but wherever he was known.

9th. That during the session of 1831–2, in which Dr. Pierson was absent, but when Drs. Drake and Henry were connected with the school, although the aggregate price of the tickets was advanced from $ 64 to $ 100, the number of students was greater than it had ever been before; and that, in the succeeding session, when Dr. Pierson was restored, and the price of tickets was but $90, the number of students was reduced to about one half of what it had been the preceding year, through the mismanagement and favoritism of the said Trustees.

10th. That the said Board of Trustees have rendered the present organization of the said Medical College defective, by the improper abolition of the chair of the Institutes.

11th. That some of the present Professors are manifestly deficient in qualifications; and that the whole, taken together, do not form such a body of teachers as to entitle the School to the confidence of the profession and of the public at large, and that whereas, from the population, water and other communications of the city of Cincinnati; its central situation in the West, and the opportunities which might be afforded by the Hospital and Asylum for the study of practical medicine and morbid anatomy, the said city is better calculated to support a great Medical School than any other city west of the mountains:—The said Trustees, by their partiality, prejudice, and illiberal management of the professorial department have interrupted the harmony, checked the progress, and weakened the popularity of the School committed to their care; and that it is confidently believed that by proper exertions on the part of the said Trustees, all the professorships might have been, and might now be filled with men of superior abilities and extensive reputation, who would soon render the said Medical College the second or third school in the Union, in point of numbers.

12th. That the present Board is incompetent to the charge and superitendance of a medical school, as it contains but one medical man.

13th. That an influential member of said Board of Trustees is an avowed and practical advocate of what is generally termed the Thompsonian, or steam system of medicine, and should not, therefore, preside over a College for regular, scientific instruction, nor be allowed to subscribe the diplomas of its graduates.

14th. That the abolition of the chair of the Institutes, referred to in specification 10th, was totally uncalled for, and had solely for its object to remove Dr. Pierson from that chair, for which he had confessed hmiself unprepared, in order that a pretext might be raised for re-instating him in the easier chair of Materia Medica.

15th. That the said Trustees have mismanaged the Library, and greatly limited its usefulness, by omitting and refusing to establish a library ticket, to be purchased by physicians and by students.

### Charge Second.

That the Board of Trustees of the Medical College of Ohio have been guilty of gross negligencc in the management of the funds of the state committed to their hands for the benefit of the said College.

*Specifications.*

1st. That in planning the edifice erected at the expense of the state for the Medical College of Ohio in Cincinnati, in the year 1826, the rooms destined for chemical purposes were so badly and improvidently planned, that in 1832 the said Trustees were compelled to take down and rebuild the whole interior of the same, at an expense to the state of several hundreds of dollars.

2nd. That in commencing the additional edifice in 1831, belonging to the said College, the said Trustess did not, as was their duty, nor did the Faculty of the said College, ever examine the plan of the said additional edifice; in consequence of which neglect, the state was put to the charge of several expensive alterations in the progress of the building.

### Charge Third.

That the Board of Trustees of the Medical College of Ohio, contrary to their known duty, permit circulars and reports on the state of the institution to go forth to the General Assembly and to the world, which they have never seen and examined—to wit, the annual circular of the Institution dated the 27th of May, 1832; the annual report of a committee of the said Board of Trustees to the General Assembly, on the 18th of the ensuing December, and the memorial of the said Board to the General Assembly, on the —— of February, 1833.

### Charge Fourth.

That the Trustees of the Medical College of Ohio, have injured the public estimation of the said College abroad and at home, and have greatly impaired its utility and respectability, by uncalled for partiality towards men, who, as professors, had no claims upon them, and by unjust persecution of able, respectable, and unoffending individuals, whom they were bound in good faith to protect.

*Specifications.*

1st. In fraudulently endeavoring to break up a rival institution, by detaching some of its professors from their pledges to their associates.

2nd. In granting an indulgence to Dr. Pierson, altogether unprecedented, in reference to a chair purely *intellectual.*

3d. In countenancing and encouraging slanderous statements in reference to the lectures of Dr. Henry, the Professor of Obstetrics, without, on any occasion having manifested the least disposition to defend his character, or even to give him prompt notice of the existence of such statements.

4th. In encouraging or permitting the attack which the Township Trustees made on Drs. Drake and Henry in January, 1832.

5th. In conniving at and encouraging the attack make upon the Professor of Obstetrics, in the month of February, 1832, by the Township Trustees.

6th. In unjustly retaining for seven days the letter of the Township Trustees, impeaching the character of a Professor of the College, without communicating to that Professor, or to the Faculty, the letter, or the fact of its existence.

7th. In suffering the false statement of the Township Trustees that "they had never instituted an inquiry into the subject," to pass without contradiction.

8th. In having contemplated, early in the session, the abolition of some of the professorships which they had established during the preceding summer, against the wishes of the students and the public, for the purpose of unjustly depriving a professor or professors of their offices.

9th. In having extorted or frudulently obtained the consent of a majority of the Faculty to the proposed reduction of the professorships—whereby their contract, compromise, or consolidation during the preceding summer, with the professors of a rival school, was openly violated, to the bad name and manifest injury of the College over which they presided.

10th. In retaining one Professor, from private family consideration, whose chair they had abolished, and expelling another, without cause assigned, whose chair was preserved, and that in opposition to the expressed wishes of a large majority of the class then in attendance.

*Charges against the Faculty of the Medical College of Ohio at Cincinnati.*

1st. That the said Faculty refused and omitted to establish a hospital ticket, for students who wished, during the eight months in which the said Medical College was not in session, to attend the practice at the Hospital and Asylum at the city of Cincinnati, while they admitted their private pupils gratuitously, thus rendering these public charities a *bonus* to young men to study with them, and deprived the College of a considerable source of revenue, specifically pledged in the law establishing these Infirmaries, for the purchase of Books, and Philosopical apparatus for the said Medical College.

2nd. That the said Faculty, whose duty it was, have neglected to give proper attendance upon the lunatics of the Asylum, except when they were ill of some malady of a common kind, superinduced upon their insanity, alleging that the building provided by the Township Trustrees and the general arrangements for the care and keeping of the lunatics, were such that no hope of curing their lunacy could reasonably be entertained; but, at the same time, that the said Faculty neglected, as was their duty, to report the same to the General Assembly.

3d. That the said Faculty have neglected and do still neglect to render the Hospital and Asylum a school for the study of morbid Anatomy, to the extent which it is practicable, whereby they have lost, and still do lose to the said College, the chief advantage which these Infirmaries might be made to confer upon the pupils thereof.

4th. That the said Faculty have sought to discourage private lectures to students of Medicine, both in summer and in winter, by others than themselves, whereby the attractions which Cincinnati might exert upon students have been diminished, just so far as the said Faculty have succeeded in the suppression of such lectures: That this is the policy which the said Faculty have pursued in defiance of the example of the best regulated medical institutions of other places; the more a Medical College can surround itself with private lectures, the greater being its popularity and patronage.

5th. That Dr. Staughton, as Dean of the said Faculty, failed to call a Faculty meeting, as was his duty, when in February, 1832, the Trustees of the College sought an approval of their scheme of reducing the number of professorships to six.

6th. That the Surgical Professor of the said Faculty has been guilty of neglect or mal-practice in the Hospital in the two cases of compound fracture, within six months of each other, in both of which, death apparently took place in consequence of said neglect or mal-practice.

7th. That the study of Practical Anatomy has not been conducted in a manner to offer the greatest inducements to medical students, or equal to the advantages which the said College might have afforded.

8th. That a majority of the said Faculty attended the last semi annual meeting of the First District Medical Society, and by their personal influence, dishonorably exerted, endeavored to obtain from said Society a vote of approbation of the conduct of the Trustees of the said College.

*Additional Charges against the Trustees and Faculty.*

1st. That in the late annual report to the General Assembly of Ohio, the Trustees and Faculty of the Medical College of Ohio have knowingly put forth false statements as to the number of pupils in attendance upon the lectures, as to the causes of their diminution from what they were the preceding year, and as to the causes of the difficulty of prosecuting *post mortem* examinations in the Hospital and Asylum.

2nd. That during the time when the Professor of Clinical Medcine was *de facto* a member of the College, one of the said Trustees put forth a false, insiduous, and anonymous publication in the newspapers, designed to reconcile the public mind to the resignation which the unjust policy of the said Trustees had rendered unavoidable to the said Professor.

3d. That after having in the most unjustifiable manner compelled the Professor of Clinical Medicine to resign his chair, they proceeded to justify their conduct by traducing his character; thereby tarnishing the good fame of the institution, and converting his friends into enemies of the school, to the injury of its prospects.

4th. That the Trustees and Faculty of the said Medical College have attempted to prejudice the fairness of the present Legislative investigation, and to screen their own doings from too close a scrutiny, by falsely and willingly attributing to the influence, maliciously exerted, of a single interested individual in the city of Cincinnati, the dissatisfaction of the physicians throughout the state, with the management of the said College; alleging that said physicians are the subservient tools of the said individual; thus giving just cause of offence to the profession in Ohio, and converting them into enemies of the said Medical College.

Signed, on behalf of the late
Third District Medical Scociety
of the State of Ohio,
JOSHUA MARTIN, M. D.
Late President T. D. M. Socy.

*Cincinnati, April* 12, 1833.

The Commissioners continued in session for nearly three weeks, and examined between 40 and 50 witnesses. In the succeeding December, they made their Report to the General Assembly; and accompanied it with several hundred manuscript pages of testimony. How far this testimony went to sustain the Charges, they did not say; but leaving that matter to the General Assembly, recommended an enlarged Board of Trustees and a new Organization. The Report and the testimony were referred to a Select Committee, who, after some weeks, made a Report on the same; which was promptly adopted with one dissenting voice. *It was not discussed, nor was the testimony to which it related ever read before the Senate.* Thus that honorable body gave its vote in total ignorance of the testimony, which, at a heavy expense to the state, it had ordered to be taken. Why it should have done so, is and will, perhaps, remain unexplained, but the fact deserves to be recorded, as a part of the legislative history of the institution.

From beginning to end, the Report is laudatory of the Trustees and the various Faculties—commendatory of the establishment—and abusive of the Editor of this Journal, whose name is introduced more than twenty times! It is certainly quite a distinction to be made thus the subject of an elaborate and lengthened state paper, by a Committee of the most dignified branch of our Legislature! Towards the close of this Report on the conduct and character of "Drake," as the

Committee courteously designate him, they declare "*That the Charges against the Agents of the State are* NOT SUSTAINED *by the evidence;*" consequently the "Agents of the State" are acquitted, and *one* of the fifty witnesses singled out for calumniation, within the very walls, where he once personally made those explanations to the General Assembly, which led to the establishment of both the College and the Hospital!

What influence could have induced the Committee to palm upon the Senate a declaration so *foreign to the fact*, as that in the quotation just made, will perhaps never be found out. The most charitable conclusion would be, that the Report was prepared for them in Cincinnati. If they wrote it themselves, they either omitted an examination of the testimony, or did that which is less excusable. This is a grave accusation, but the Editor proposes to make it good by a reference to the unread and unpublished evidence now buried in the Archives of the Senate. He will proceed then to state what charges *were* sustained, by that testimony.

Before proceeding, however, to this object, the Editor would remark, that it is difficult to understand precisely the extent of the assertion, that the "charges against the agents of the State have not been sustained." The sentence is artfully framed. First. The Faculty are not the agents of the State, and consequently are not embraced in this sentence of acquittal; and still, no doubt it was intended by the Committee that it should be so construed as to include them. Secondly. Do they mean that *all* the charges, or only a part of them, were not sustained? If the former, the assertion is one, perhaps, unequalled in the annals of legislation, for recklessness of fact. They could not *say* that *all* the charges were not sustained, but desirous of making that impression, without committing themselves, they say the "charges are not sustained." The Editor does not pretend that *all* the charges were supported, but he knows that many of them were, and will proceed to show it. He will refer to them by numbers, to avoid repetition.

In reference to the first specification of the first charge, it appeared in evidence, that the Trustees supported Dr. Cobb, of the state of Maine, to the chair of the Theory and Practice of Medicine, within a year or two after his graduation, when he was a very young, and an entirely inexperienced physician; and that he himself, from a consciousness of want of qualification for that practical chair, insisted on being transferred to another. Although the Committee say, the charges were not sustained, they admit this, and artfully omitting, in their reasonings upon it, any reference to the age and inexperience of the youthful incumbent, observe, that they do not believe, that because a man is born in New England he is disqualified from being a Professor in Cincinnati. If the Committee had been candid and intelligent on this matter, they would have admitted, first, that no young physician, with the ink not yet dried on his diploma, was qualified to teach the *practice* of medicine; and secondly, that the selection of a professor of the practice of physic from the state of Maine was injudicious, because no students could be expected from that quarter, and because its diseases differ in many respects from those of the West.

In regard to the second specification, it was proved, that the Trustees, as late as February 1831, had said to the Legislature, by a committee, that in no school, either East or West of the mountains, had the fees of tuition been put as low as in this. Here again the charge was sustained; and the Committee go on to justify the Trustees and Faculty. They cannot see in this underbidding, any consciousness of inferiority on the part of the Faculty; they cannot see, in the fact that the Transylvania School, refused, on this account, to acknowledge a course of lectures in the Medical College of Ohio, as equivalent to one in that school, any degradation; they cannot see that *poor* young men have, generally, had limited opportunities for preparatory education, and are likely to be imperfect in their scholarship and professional attainments, and, of course, not equal to the graduates of other schools, selected by the better educated; finally, they cannot see the testimony of the Editor on this point, as it was delivered, but condescending to play the demagogue, they make him say, that poor people do no credit to a school, and then put themselves forth as the friends of the poor; when his opinion was, as just expressed, that poor scholars are, in general, of necessity, so superficially educated, that they cannot reflect upon the institution in which they graduate, the same credit they would confer if their early opportunities had been ample.

The fifth specification sets forth, in the beginning, that it was the *weakness* of the Faculty of the College for eight or nine years, that led to the projection, in Cincinnati, of a rival institution, under the auspices of the Miami University. Here, again, another charge was sustained, although the Committee declare, in the close of their report, that the "charges are not sustained." The evidence in support of this charge was ample. It consisted, in part, of letters, which were verified by their authors, and sent up by the Commissioners to the General Assembly, and of course were before the Senatorial Committee. The Editor will introduce some portions of them here. The testimony of Dr. Staughton and the Editor, who made a personal application to the Trustees of the Miami University, to establish a Medical Faculty, and of Dr. Eberle, who was one of its original members, may, perhaps, be considered conclusive on the point—that it was the weakness of the Medical College of Ohio, that invited the establishment of a rival school.

In his testimony, the Editor stated specifically, that but for the generally acknowledged insufficiency of the College, and the fact, that its Trustees had refused to re-organize it, he should not have attempted to project another. That Dr. Staughton thought in the same way, and predicated his actions of that conviction, will clearly appear from the following extract of a letter, written to a friend in the Eastern States, whom he solicited to unite in the enterprise. This letter was dated October 14, 1830, several months after Dr. Staughton's arrival in Cincinnati. It was verified by himself before the Commissioners, and the original is now in the archives of the Senate.

"Before this letter is placed in your hands you will have received from my friend, Dr. Drake, a communication on the subject of your removal to this city. The early and sincere affection which has united us, prompts me to present the subject to you in the plainest manner possible. It is a *notorious fact* that the Medical College of this State, is in the hands of professors in *no way competent* to the discharge of the sacred trust reposed in them. This you know, and this I knew *before* I crossed the mountains; but I had *no idea* that it was *possible* for any men who held the name of Professors, to be so dead to *public opinion*, so *insensible* to those longings after professional *reputation*, which I once thought must necessarily be within the bosom of *every* public teacher. I would that I could find time and space to enumerate instances in proof, but it is enough that the *profession of the city feel* that the Medical School, in its *present condition*, is a *disgrace* to the State—it is enough that the community *feel* it. 'Sir,' said our most distinguished lawyer (Mr. B.) to me not long since, 'I feel *degraded*, as an inhabitant of Cincinnati, whenever I think of the manner in which our Medical School is conducted.'

"The question naturally arises, why then does not the Legislature look into the matter, and re-organize the School. Political motives, of a sectional character, have prevented, and most probably will prevent, till some public demonstration is made. Dr. Drake will explain this point more at large than I can do in a letter.

"Dr. Drake, with whose name and history you are perfectly familiar, has been looked up to by those medical men in the valley of the Mississippi, who have felt the *low estate of the Ohio Medical College* as the one on whom their hopes of a *better* state of things rested. From the intimacy which has subsisted between the Doctor and myself during my residence, I have no hesitation in stating, that I know of no one in the United States, so well qualified to undertake the foundation of a Medical School. To high and commanding talents, the Doctor adds a consummate knowledge of men. Naturally eloquent, he has cultivated his powers to an extraordinary degree. He possesses what both you and I prize not a little, amenity of manners, and kindness of heart. The great object has been before him a long time. With undaunted perseverance he has at last obtained the privilege of nominating the Professors of a medical school, a branch of the Miami University. I cannot enter into a history of this highly respectable institution. Its endowment of 22,000 acres of land will render it the *richest* seminary in the United States.

"Cincinnati is, beyond doubt, the place for a medical school. There *must*, in the nature of things, be a great college here. The present is the moment for the commencement. With the talents that it will be the Doctor's aim to enlist in the undertaking, there cannot be the slightest shadow of a doubt as to the *immediate* success. The proposition that will be made to you, must be sufficient to convince you of the confidence of the friends of the cause.

During the sojourn of the Editor in Philadelphia, when, as Dean of the projected Miami Faculty, he was seeking for colleagues, be received several letters from Dr. Staughton, designed to aid him in that public object. Most of them urged on him the claims and qualifications of Dr. McDowell, then a private lecturer on Anatomy in Cincinnati.

In these letters Dr. S. expressed the results of his observations on the existing Faculty of the College. The following, from a letter now in the archives of the Senate, may serve as a specimen. It is dated November 14th, 1830.

"McDowell astonishes us:—His first lecture far, very far surpassed the College lectures; and on Friday I heard him give a lecture on the temporal bone, which was equal to any thing I ever heard on the subject.

"As to the College lecturers—Pierson was puerile to the verge of silliness. Smith's ideas were wholly from Abernethy, save and except a beautiful simile, between the functions of the system and the inhabitants of a city, which was emphatically *his own.* Slack lectured on galvanism, as a mode of curing all diseases, and on the divining rod!! Whitman I did not hear. The broad slang and low vulgar tales of Moorhead, reminded me of some of the discourses I had heard in the vagrant cellar of the Philadelphia Alms House. Cobb, for an introductory, read a translation of a description of a painted pasteboard man he bought during his seven-day sojourn in France, and after that the report of the committee of the House of Commons in England on the subject of dissection."

Dr. Staughton was afterwards a professor in the College, and that the distant reader may know whether his opinions, relative to the Faculty of 1830, are entitled to credit, the Editor will here reprint the following Resolution of the Board of Trustees, unanimously adopted on the occasion of the death of that Professor, August 9th, 1833, after he had been two years a member of the school.

"*Resolved,* That this Board learn with deep regret of the decease of Dr. James Staughton, Professor of Surgery in the Medical College of Ohio: a gentleman whose talents, acquirements, and moral and professional integrity, had secured to him the esteem of the public, and endeared him to the institution with whose prosperity he was so closely and warmly identified."

The following extract of a letter to the Editor, as Dean of the Miami Faculty, written by Dr. Eberle and dated Philadelphia, March the 4th, 1831, will disclose his convictions of the weakness and inefficiency of the Faculty of the College as it then existed. It refers to an abortive effort of the Trustees of the College, to move the Legislature against the Miami school, in the preceding winter.

"The interest manifested in our behalf by your *medical* friends in Cincinnati and elsewhere, and the evident destitution of such support on the part of our enemies, is especially encouraging, as it betokens a kindly feeling towards our enterprize in the mass of the profession—the most certain evidence at once of the necessity and propriety of the effort we are making. The fact, too, of so large a portion of their own pupil's signing our memorial, speaks loudly against the present school and in favor of our own undertaking. But the climax of damning circumstances, is the blasting report of the legislative committee—a formal acknowledgement and public declaration that the existing institution is wholly insufficient. Weak men should never provoke a contest; an ungenerous and unmanly opposition almost always defeats its own unhallowed purposes.

On the 31st of the same month, Dr. Eberle in another official letter, still speaks in a language that clearly indicates that his hopes of the success of the projected Miami School, were founded on the low estate of the existing institution.

"I have lately conversed with several gentlemen from Ohio—merchants in some of the interior towns; and they have uniformly spoken in the most encouraging terms of the enterprise we are engaged in. It does indeed, appear from what I have learned, that the present Faculty of the Cincinnati College, are by no means very formidable opponents."

In the succeeding winter, 1831–2, after several months acquaintance with the professors of the College, Dr. Eberle found his previous impressions in regard to one of them, Dr. Moorhead, the Professor of Theory and Practice, fully confirmed, for he declared to Dr. Martin, whose testimony went up to the Senate, that Dr. Moorhead was "30 years behind the profession."

It was in evidence, then, before the Senatorial committee, that those who projected the rival School in this city, were encouraged to do so, by the low reputation of the Medical College of Ohio in 1830; although it was then quite as high, perhaps higher, than it had ever been before.

The Trustees themselves, indeed, afforded a practical manifestation of concurrence in the same opinion, for before all the Professors of the new or rival School had reached Cincinnati, they displaced from the College one half of the incumbents, whom, in defiance of public opinion, they had cherished from 1823 or '24, a period of seven years.

Do not these different facts, then, clearly substantiate the heavy charge, that for many years the Trustees had kept in office, to the great injury of the institution, and of the interests of the profession, an incompetent Faculty? And yet the Senatorial Committee make the sweeping declaration, that the "charges against the State's agents were not sustained by the evidence!"

If no others were sustained, it is manifest that this was; and if no other had been, this alone would have kept a Committee, who respected the interests of the State, the rights of individuals, or their own character, from the sweeping verdict of approbation and eulogy which was reported to the Senate, *through* those to whom that body confided this matter.

Why! this was the very essence of the complaints of the medical profession, and of different societies in the State—that the Board had appointed incompetent men, who could exert no attraction on the pupils of the Valley of the Mississippi, who never investigated, who never wrote, who were unknown abroad, and *un*respected at home; and that it clung to them, until the establishment of a rival school, invited by their incompetency, compelled the Trustees to enter on a reform, or see the College utterly prostrated.

To this reform, the reader who may be inclined to pursue the subject, will now please to direct his attention.

On the 9th of April, 1831, Col. Davies, a member of the Board, and the brother-in-law of professor Pierson, presented his memorial, already mentioned, to the Senate, praying for a law to estop the Miami University in establishing a medical department. The Editor of this Journal, then returning from Philadelphia, happened at Columbus, and the next day presented a remonstrance. The committee to whom they were both referred, reported in favor of a new and enlarged board of Trustees, and a re-organization of the faculty, which report was laid over till the first Monday of the ensuing December, and never called up. Before the Committee had reported, Col. Davies proposed that Dr. Eberle, Dr. McClellan, and the Editor, should leave their Miami colleagues and go into the College, with Drs. Pierson, Cobb and Moorhead. The Editor told him that he was under obligations, expressed and implied, not to abandon any of his colleagues, that he would respect the pledges he had given, and that, unless *Drs. Mitchell, Staughton and Henry were provided for, he would never consent to go into the College.* In the Spring the Trustees and Professors of the College made an unsuccessful effort to arrest the Miami organization, by a resort to the writ of *quo warranto.*

On the 21st of June, the Trustees declared all the Professorships vacant, and elected Dr. Mitchell to that of Chemistry, Dr. Staughton to Obstetrics, and the Editor to the Institutes, a chair created in 1827, but never filled. This left Drs. Eberle, McClellen, and Henry unprovided for; and as the Editor had declared, in Columbus, three months before, that his pledges to his colleagues put it *out of his power* to leave any of them behind, it was evident that he was offered the place, merely to amuse and delude his friends; with the certain knowledge that he *could* not accept it. As was his duty, he apprized the venerable President of Miami University of this offer, and received for answer, that "*he must on no account whatever desert any of his colleagues,*" but was told, as the opinion of the President, that if the whole of them were offered seats in the College, the Trustees of the University would be satisfied that they should accept.

The Trustees of the College, however, had no such intention. Their design was to detach Drs. Mitchell and Staughton. They believed, that although the Editor had refused to abandon those professors in the winter, *they* could be *induced* to abandon him; and the Trustees were not mistaken in the estimate they formed of the character of those persons, as we shall presently see. Finding, however, that Drs. Mitchell and Staughton were slow in coming over, and that they were anxious to have Dr. Eberle in the school, the Trustees determined not to re-elect Dr. Smith, but to give his chair, Surgery, to Dr. Staughton, Obstetrics to Dr. Pierson, and Materia Medica to Dr. Eberle; Dr. Cobb was re-elected to Anatomy and Dr. Moorhead to the Theory and Practice. This left Drs. McClellan and Henry unprovided for; but the former was *willing* to remain in Philadelphia, and it was only necessary to secure a place for the latter. At this critical juncture, the Editor received a letter from Dr. Eberle, saying, that *Dr. Mitchell* had just written to him, that *he* and *Dr. Staughton* would enter the College, and leave Dr. Drake behind, if he, Dr. Eberle, would unite with them. This letter was verified and sent up to the Senate. Finding himself thus treacherously deserted, by those whom, but a few months before, he had peremptorily refused to abandon, the Editor had no resource, but to see the Miami Faculty, decomposed about him, or to suggest an extension of the Faculty, of the College from seven to eight professorships. This led him to propose the chair of Clinical Medicine, and to offer to take it himself, and yield the Institutes to Dr. Pierson, that Dr. Henry might have Obstetrics, the chair which he then held in the Miami School. The Editor would have preferred the Institutes to Clinical Medicine, but Col. Davies, the brother-in-law of Dr. Pierson, was opposed to the extension, Dr. Pierson had returned to his residence in New York, and the Editor felt convinced, that if he proposed such a limited and doubtful professorship, for the relative of a governing member of the Board, the whole plan of consolidation would fail; and, two of the members of the Miami Faculty leaving it, that project would be at an end. Therefore it was, that the Editor, proposed to take that chair himself, and that it should be left *optional* with the students to take or omit his ticket, expressing to his friends, that if he found the professorship too limited he would resign at the end of the first session. The Trustees were now concluded; but it was with great reluctance they confirmed the arrangement.

Dr. Pierson's appointment to the chair of Obstetrics, having been revoked in two days after it was made, was not sent to him. When the letter of the Secretary, informing him of his transfer from Materia Medica to the Institutes, reached him, he declined accepting the latter, unless the Board would allow him a year for preparation.

The reception of this lettter was an important event in the progress of the school. The Board must either grant or refuse the request. They did not want eight professors. Dr. Pierson resided in New-York, and they were under no necessity of allowing him a year for preparation. If they did not grant him that favour, the union of the Institutes with Clinical Medicine, would form a good professorship, and the incumbent of the latter, who had been in some degree made a rallying point by those physicians of Ohio, who had labored to reform the College, would have been enabled to remain in it. But the Board did not wish that; and granted the request. The Session commenced, and the Institutes, *pro tempore*, were united with Clinical Medicine. From the limited extent of the wards of the Hospital, it was soon found, that Clinical Medicine could not be sustained as a separate chair. The students almost unanimously desired the two to remain united; but the Board clung to Dr. Pierson, and the Professor of Clinical Medicine, on whose application, twelve years before, both the College and Hospital, had been chartered by the Legislature, was compelled to resign. Such is the abridged history, of the consolidation of the two schools, as it was delivered in evidence before the Commissioners, and sent up to the Senate.

The resignation of the Professor of Clinical Medicine left seven professors, and called for nothing but the abolition of that chair. The Board, however, proceeded likewise to abolish the chair of the Institutes, then they displaced Dr. Henry, in whose favor nearly all the class had petitioned, and who was a native of the West; transferred Dr. Moorhead to his chair, gave the Theory and Practice to Dr. Eberle, and thereby opened the way for the restoration of Dr. Pierson to Materia Medica, after he had been eight months preparing himself for the Institutes! Thus two Western Professors were sacrificed, and one important chair abolished, to bring back to Materia Medica, a physician resident in the city of New-York, because he was the brother-in-law of an influential member of the Board, who was on *every* Committee which had these complicated and sinister movements in charge. The evidences of all of this, again, were before the Senatorial Committee, when they declare in opposition to the facts, that the "charges against the states' agents were not sustained."

But the Senatorial Committee in their partizan zeal, have gone still farther. They have asserted that Dr. Pierson received his appointment, with the express stipulation, that he should be absent; which means, that he should stay away to prepare himself;—but no such stipulation was made or sent to him with his appointment, and no such evidence was before them. Honorable men, whether in a private or public capacity, ought to feel desirous to explain away or correct such an error.

It was these various manœuvres, that stirred up anew, the members of the Third District Society, and the General Medical Society of the state, and led to the memorials on which an inquiry into the conduct, management and actual condition of the Institution; but after it was made at an expense of many hundred dollars of public money, the members of the General Assembly, never read the testimony, and voted that all had been properly done and that all was well. How far the facts that have been detailed, go to show partiality and favoritism, in the Board, every reader must judge for himself.

Immediately after the resignation of the Professor of Clinical Medicine, Dr. Pierson's brother-in-law, wrote and published in one of the papers of the city, a piece designed to show, that the College which then existed, was not the same corporation which had been founded by that Professor; and in two or three months, an advertisement, signed with the names of the President and Secretary of the Board, appeared in the gazettes, asserting the same thing. All the facts, however, demonstrated the reverse, and the Commissioners declared against this unjust and ridiculous pretension. It was set up to reconcile the friends of the Professor of Clinical Medicine, to the course of policy which had compelled him to resign.

The advertisement just mentioned, deserves a further notice. It praised the Faculty and called on the community to extend its confidence to them; it was in short a kind of eulogium and certificate of character; and might have been of service to them in after times, if it had not, unfortunately, come out in evidence before the Commissioners, that neither the Board of Trustees, nor the President and Secretary, whose names were appended to the paper, had ever seen it! The Faculty had got it up themselves, and sent it forth to the world under the signatures of persons who had not even heard of it, till they read it in the newspapers! This is one of the examples cited in the fourth charge. Another was the annual report of December, 1832, to the General Assembly, on the state of the Institution, which, as appeared in evidence, was never seen by the Board, and, in fact, but by one of the Committee of two, whose names were attached to it! Another instance of a similar kind, referred to in the same (fourth) charge, was a printed address to the Legislature in the following February, 1833, containing a great variety of misstaments, avouched by the names of the whole eleven members of the Board, including one who was then a Senator in Columbus; although it was proved before the Commissioners, that it had never been read in the Board, and was not even drawn up by a Committee appointed by that body, but by some individual member, and the names of the Trustees affixed to it, without a part of them at least, ever having heard it read! All this was known to the Senatorial Committee, and still, they have the pliancy to tell the Senate, that the "Charges against the state's agents are not sustained by the evidence."

The restrictions which the limits of this Journal impose, require that this article should not be extended much farther, and many other charges, therefore, can only be referred to.

The 9th, 13th and 15th specifications under the first charge, were fully sustained; and in regard to the 9th, it was shown, that the Faculty had made a deceptious return to the Legislature of the number of pupils for 1832–3.—Still, the "Charges against the state's agents were not sustained by the evidence."

Both the specifications under the second charge were fully sustained. The third charge has been already mentioned.

The first and second specifications under the fourth charge, have been likewise commented on. The third, fourth, fifth, sixth and seventh, specifications under the same charge, were amply supported; and the eighth, ninth and tenth, were rendered highly probable.

The first of the charges against the "Faculty of the College," p. 629, was established in the most unquestionable manner. The second, third and fourth were supported by a variety of of testimony. The fifth and eighth were proved.

The whole of the "Additional Charges against the Trustees and Faculty," p. 630, were supported by testimony the most conclusive. The two first have been already referred to. The third and fourth were made out by irrefutable proofs.

For the truth of all that is here asserted, the Editor solemnly appeals to the testimony taken down by the Stenographer of the Commissioners, and deposited in the archives of the Senate—unread by that honorable body, and placed beyond the reach of the people. It may seem strange to the readers of this Journal, who are physicians, that the Senate could thus lose sight of its dignity and duty; but a fact or two will dissipate the mystery.

1. An old and influential Senator, himself both Physician and Lawyer, is a Trustee, and could of course tell the Senate how to act; if, indeed they could have been so deficient in courtesy, as to do or propose any thing that might affect his feelings as a *nominal* member of the Board. 2. The country members of the General Assembly leave the affairs of the College, to the decision of those from the city where it is located; and it does not require Major Downing's slate and pencil, for them to calculate the difference, *on election days*, between the votes of eleven Trustees and six Professors, with their friends and dependents, and *one* man,—unsustained by any faction, unallied to any cabal, and occupied in writing dull editorials for the Western Journal.

Towards the close of their report, the Senatorial committee observe that,—

"That they have no doubt of Dr. Drake's qualifications and believe he is capable of doing honor to any Medical School. But they are compelled to say *from the facts disclosed in this case*, that it is extremely problematical, whether peace and harmony could prevail for any length of time, in a Medical Institution with which he should be connected, unless it was entirely under his controul and management."

How must the different Faculties to which Dr. Drake has belonged, congratulate themselves that they should have got rid of him, before their "peace and harmony" were broken up! That he destroyed the "peace and harmony" of none, was in evidence before the Senatorial Committee. That he had been *twice* a member of Transylvania University and lived in "peace and harmony" with the Faculty and Trustees, was among the "facts disclosed in this case:"—That he had lived in "peace and harmony" with the Faculty of Jefferson College, in Philadelphia, and after his resignation, was earnestly invited to return and assist in a reorganization of the School, was among the "facts disclosed:"—That Dr. Eberle, after he had left the Jefferson School, in writing to a friend, spoke of Dr. D. as "a most worthy and honorable individual, who in the whole course of his life had maintained the character of a high minded, honorable, upright and intelligent man," with whom he was anxious to be again associated, the projected Miami School, was among the "facts disclosed:" —That during the brief existence of that Faculty, he lived in "peace and harmony" with its members, was among the "facts disclosed:"—That during the whole time he belonged to the College, after the consolidation of the two Faculties, he lived in "peace and harmony" with them, was among the "facts disclosed." If the Senatorial Committee chose to express the opinion which they did, without referring to the "facts disclosed," he certainly would make no objection; but when they pretend to base that opinion upon the "facts disclosed," he is at liberty to say, that by a slip of the pen, they have written the opposite of the conclusion which the "facts disclosed" require. No doubt the members of the Committee are all honorable men in private life, and would not willingly pervert or suppress the truth, for the purpose of calumniating a fellow citizen; but who is so simple minded as to suppose, that legislators and politicians are expected to adhere to the "facts disclosed?"—If this were done, the "peace and harmony" of factions would soon be annihilated.

Post Script.

As a specimen of the gossip with which the Trustees and Faculty amuse the General Assembly, in their annual Reports on the progress, state and prospects of the College, the Editor gives the following extracts from those of December 1832 and 1833.

Report of 1832. " *Carthago est delenda.* The Great Apollo has issued his behests from his tripod in this city, and his little but faithful court of sub deities are raising the shout and pointing their shafts at the devoted heads of the Trustees and Faculty of the Medical College of Ohio"!

It was certainly most humane in the Senatorial Committee, to cast the ægis of the State before the devoted heads of these worthy gentlemen whom Dr. D. is always persecuting.

Report of 1833. "But the cause which has operated with the greatest effect in producing this result, is, the continued efforts of the enemies of the Institution, to injure its reputation abroad, and to persuade students who had determined to enter this College to change their determination, and repair to other Institutions. It is a fact, of which there is ample proof, that several students who had arrived in this city for the purpose of joining our Institution, was told, that it was in a sinking condition, and that they would, if they consulted their own interests, leave the city and enter the College at Lexington. This advice, we know, had its intended effect in several instances. We also know, that letters have been written by medical men in this city, to others in Kentucky, misrepresenting this Institution, and advising them to send their students to other schools. Thus it appears, that these persons by propagating slander, prevent the collection of a large class, and afterwards resort to *that fact,* in proof of the slander. Had it not been for these, and similar measures, there is reason to believe that the class, at this time would have amounted to two hundred."

A LETTER FROM PROFESSOR MITCHELL.

To the Board of Trustees of the Medical College of Ohio. Gentlemen:—It is proper that I should communicate to you the following information. Sometime in the first or second week in November, of the present year, Mr. William J. Barbee, a student of Medicine from Paris, Kentucky, stated to me, that his preceptor Dr. Telford, a few days or weeks before he, (the said Barbee,) came to Cincinnati to attend the lectures, had received a letter from Dr. Drake, which represented the prospects of a class in the Medical College of Ohio, for the approaching session, as very doubtful, and *advised Dr. Telford to send his pupils to the Lexington school.* I have further to state, that two students from Washington, Kentucky, who had come here for the purpose of deciding whether they would become pupils of this school, or go to Lexington, stated to me, as one objection to remaining here, that it was currently reported in their town, that a course of lectures in this Institution would not be credited in another school. It was in the presence of these students, that Mr. Barbee made the statement above referred to. The information which the Washington students obtained, touching this difficulty, satisfied their minds, and they immediately joined the class. It is proper here to state, that the report put in circulation at Washington, has no foundation in truth, although its tendency to prevent students from coming to the Medical College of Ohio, must be obvious to every one. From my conversation with the Washington students, I was satisfied that they would have gone to Lexington, if they had not resolved to take Cincinnati in their route for the purpose of obtaining information.

Very respectfully,

THOMAS D. MITCHELL, Dean, &c.

Cincinnati, Dec. 4th 1833.

FROM PROFESSOR MOORHEAD,

To the Secretary of the Board of Trustees of the Medical College of Ohio.

Cincinnati, Dec. 4th 1833.

Sir, Some time about the middle of October last, three students from the South, came to this city, for the *purpose* of entering the Medical College of Ohio. They took their temporary residence at the Tavern of Mr. Heckewelder, while there, they were visited several times by Dr. Drake, to whom, I understood, they had letters of introduction. After these visits, they left the city and went to the school at Lexington, with letters of introduction from Dr. Drake. For the truth of this statement, I refer you to Mr. Heckewelder himself, from whom I received the information.

To the Secretary of the Board of Trustees.

I am, Sir, very respectfully your obedient servant.

JOHN MOORHEAD.

FROM PROFESSOR COBB.

To the Trustees of the Medical College of Ohio.

Cincinnati, Dec. 4th, 1833.

Gentlemen.—I was lately informed by Mr. Nathan Guilford, that a Mr. Ford, of, I think, Georgetown, Kentucky, on his way to this city, met at the tavern where he spent the night, three or four Medical students, who were going to Lexington to attend the lectures. They informed him, that they had just visited Cincinnati for the purpose of attending the lectures in the Medical College of Ohio, but that Dr. Drake advised them to go to Lexington, alleging that the Medical College of Ohio, was in a sinking condition, and that there would not be 25 pupils to attend the lectures the ensuing session.

The Honorable the Board of Trustees of Medical College Ohio.

JEDEDIAH COBB.

Now when the Commissioners of inquiry were in session, Dr. Martin sent to them an affidavit, which contained some things communicated to him by Mr. Ellsberry; but the Committee of the Board of Trustees expressly objected to them because they were *hearsay*, declaring that the Trustees could not consent to be condemned on hearsay testimony. It seems, however, that the honorable Board think that kind of testimony good enough for Dr. Drake, even when the witness, unlike Dr. Martin, who quoted a member of the Senate, is unable to give the name of his informant. It is edifying to see respectable men involve themselves in such inconsistences.

But these certificates demand and shall receive a different kind of notice.

The letter of the Editor, to Dr. Telford, a former pupil, of Troy in this State, not Kentucky, was written October 22d, only six days before the lectures in the Medical College of Ohio commenced, and was in reply to one from that gentleman, dated September 18th. Not having kept a copy of it, the Editor has sent to Dr. Telford on the occasion, and will here present the following extract from his reply:

"The case of Wm. J. Barbee was simply this: Some time last summer, his mother wrote me, asking my advice where, in the West, he had better attend a course of lectures, and desiring me to obtain your opinion. This, and nothing else, gave rise to the correspondence. I frankly state to you, that all the circumstances of the case considered, I advised Mr. B. to go to the Lexington school. I then thought, and now think it the best school, and I so stated to you *my opinion*, not dreaming it was to be made a public matter, and the ground of a charge against you. At your request, I here copy (word for word) all in your letter of the 22d October, relating to the Medical College of Ohio.

'It is generally believed here, that the Commissioners of enquiry into the state of the Medical College of Ohio, will report in favor of enlarging the Board. Should they do so, and the Physicians of the State think proper to exert themselves with their Representatives, important results may follow.

'As yet, I believe very few pupils have arrived here, and two who had been clamorous friends of the reigning dynasty have lately set off for Philadelphia.' This is every word of the correspondence."

The letter in answer to Dr. Telford's, was the only one which the Editor wrote to any person on that subject, during the vacation of that year. Further, Mr. Barbee denies the most important part of Professor Mitchell's certificate:—

Cincinnati, Ohio, March 21st, 1834.

Dr. Drake,

Sir,

I wish to say that I never stated to Dr. Mitchell or any other individual, as he (Dr. M.) has represented in his letter of December 4th, 1833, to the Board of Trustees of the Ohio Medical College, and published by them in their annual report, that you had advised my preceptor, Dr. Jno. G. Telford, by letter or otherwise, to send his pupils to the Lexington School in preference to that of this place; nor have I ever understood or stated that you gave such advice to any individual whatever.

Yours respectfully,

WM. J. BARBEE.

The Professor is not *quite* perfect, but he should not be blamed over much, for the sentiment of the College is, that in every thing which respects Dr. Drake, the end will justify the means. The Professor is not altogether a novice at *such* mistakes. In the summer of 1831, when the consolidation of the Miami Faculty with that of the College was a matter of negotiation, the Professor wrote to Dr. Eberle, then in Philadelphia, that the "Trustees of the College wished him (Dr. E.) to take the chair of Obstetrics," but within thirty days forgot it, and declared that he had never written any thing of the kind, nor even mentioned that chair, and that Dr. E. must be out of his senses!

The Professor attempts by implication, to show that Dr. Drake had spread a report, that the Lexington School would not recognize a course of Lectures in the Cincinnati. This was the case when Dr. D. came into the College in 1831. It was then rescinded. For the fact, that some students had not yet come to a knowledge of the repeal of that regulation of the Kentucky School, Dr. D. is to be held responsible to the General Assembly!

The Professor is really a *good* man, but a man cannot be *good* always. *Maculœ soli sunt*—the sun himself hath spots. But for one little spot, the Professor's moral character would be still better than it is.

Professor Moorhead's certificate, *happens* to contain one small truth, which is, that Dr. D. gave the Mississippi students letters of introduction; but he did not advise them to go to Lexington, he did not know where they lodged, and did not call on them. In their first visit to him, they informed him that they were on their way to other schools. When they first landed, and before they had seen Dr. D. they told Dr. Dodge and Dr. Wood the same thing. What language they may have held when questioned by grooms and barkeepers on behalf of the College, he does not know.

Professor Cobb's certificate is a choice mess of stuff for a *gentleman* to put his name to. Several students, whose names are not given, in a tavern not designated, told a Mr. Ford, who told Mr. Guilford, who told Dr. Cobb, who told the Trustees, who told the General Assembly, who told the Public,—that Dr. Drake had advised them to go to Lexington, for that the Cincinnati School was in a sinking condition and would not have 25 pupils! The whole is utterly false, whether these unknown students, a Mr. Ford, Mr. Guilford or the Professor was the original author!

It is but an act of justice to the President of the Board, to state that he refused to sign the annual report to which these various puerilities were attached.

Dr. Drake, in conclusion, wishes to say, that after he was called as a witness before the Commissioners, in April last, and delivered his testimony, he left the whole matter to the Legislature, and neither by written or spoken words, advised any physician or student, in favor of the Lexington or any other school, in preference to that in Cincinnati. On the contrary, six of his pupils were connected with the latter, a greater number than any of its professors furnished. Notwithstanding all this, however, it seems that Professor Mitchell has received intellegence that Dr. Drake's exertions kept away 88 pupils. This is truly paying a splendid compliment to his standing in the Valley of the Mississippi. While engaged in the drudgery of professional business, and remaining constantly in one town, to direct the choice of 88 students, scattered over ten or twelve states, is a moral influence which few men in the profession have ever wielded! No wonder the Faculty and Trustees, have invoked the aid of the General Assembly against this talismanic power!

With these remarks the College is dismissed till the next number.

